# Book Reviews

**Published:** 1829-12

**Authors:** 


### CRITICAL ANALYSES.

Quæ laudanda forent, et quæ culpanda, vicissim

Illa, prius, cretâ; mox hæc, carbone, *notamus.*—PERSIUS.

*On the Nerves of the Face.* Being a second Paper on that subject, by CHARLES BELL, F.R.S. (*Philosophical Transactions,* May 1829.)

THE publication of this essay gives us an opportunity of reviewing the principal experimental inquiries which have been made during the last few years into the functions of the nerves considered as organs of consciousness. The object which we have in view is to explain not merely *what* has been done, but *to whom* the credit of each discovery is attributable. It must be instructive, and may even be amusing, to trace the progressive steps by which the body of knowledge which we now possess has been gradually unfolded.

It cannot be necessary to inform our readers that the authors of the modern physiology of the nerves are BELL, MAGENDIE, and MAYO: the first well known for his fertile invention, ingenuity, and talent; a brilliant and successful lecturer, who claims, in the history of these discoveries, the first place and mention, from having, as we shall show, originated the experimental inquiries out of which has grown our present knowledge. The second celebrated throughout Europe for the boldness and originality displayed in his experiments on living animals. The third, whom it is to praise most highly to say that he bids fair, as a practical surgeon, to equal the reputation which he early acquired as an anatomist and physiologist. Let us proceed to examine what each in his turn has done. The meed which we have to give to each amounts to no inconsiderable distinction, and by us it will most assuredly be allotted with strict fidelity and justice.

More than twenty years ago, Mr. BELL devoted himself to the study of the brain and nerves. He printed, and circulated amongst his friends, a tract which is now lying before us, entitled "Idea of a New Anatomy of the Brain." In this work Mr. Bell states, as his *then* opinion, that the cerebrum, with the anterior half of the spinal marrow, and the anterior roots or fasciculi of the spinal nerves, are the organs of consciousness, of thought, feeling, and volition; that the cerebellum, the posterior half of the spinal marrow, and the posterior roots or fasciculi of the spinal nerves, regulate the secret, organic, or automatic functions of the body, secretion, growth, the sympathies of parts, and the like. We extract the following passages to prove that we have correctly represented Mr. Bell's *then* opinion.

"The medulla spinalis has a central division, and also a division into anterior and posterior fasciculi, corresponding with the anterior and posterior portions of the brain. Further, we can trace down the crura of the cerebrum into the anterior fasciculus of the spinal marrow, and the crura of the cerebellum into the posterior fasciculus."*

"The cerebellum, when compared with the cerebrum, is simple in its form. The medullary matter comes down from the cineritious cortex, and forms the crus; and the crus runs into union with the same process from the cerebrum, and they together form the medulla spinalis, and are continued down into the spinal marrow; and these crura, or processes, afford double origins to the double nerves of the spine.

"The nerves proceeding from the crus cerebelli go every where, (in seeming union with those from the crus cerebri); they unite the body together, and control the actions of the bodily frame, and especially govern the operation of the viscera necessary to the continuance of life.†

"The cerebrum I consider as the grand organ by which the mind is united to the body. Into it all the nerves from the external organs of the senses enter; and from it all the nerves which are organs of the will pass out.‡

"The secret operations of the bodily frame, and the connexions which unite the parts of the body into a system, are through the cerebellum and nerves proceeding from it."§

The partial experimental proof which Mr. Bell adduced in support of the preceding views, is the following:

"I found," says Mr. Bell, "that injury done to the anterior portion of the spinal marrow convulsed the animal more certainly than injury done to the posterior portion; but I found it difficult to make the experiment without injuring both portions.

"On laying bare the roots of the spinal nerves, I found that I could cut across the posterior fasciculus of nerves, which took its origin from the posterior portion of the spinal marrow, without convulsing the muscles of the back; but that, on touching the anterior fasciculus with the point of the knife, the muscles of the back were immediately convulsed."||

The preceding extracts agree in substance with the views which Mr. Bell was accustomed to deliver in his lectures at Windmill street, about 1813 and 14. These views have, indeed, been proved to be erroneous; but the critical historian of the physiology of the nervous system will still

* Idea, &c. p. 21. † Ibid. p. 26. ‡ Ibid. p. 27.

§ Ibid. p. 36. || Ibid. p. 22.

give to Mr. Bell the honour of having made the *first* experiments upon the double roots of the spinal nerves. What he observed in these experiments, he observed justly; but, although it was the truth, it was not the *whole* truth. The evidence which he elicited from his interrogation of nature was faithful as far as it went; but it was partial, and it was incomplete, and therefore he was led to false conclusions. The subject now slept for a while.

In 1821 Mr. Bell published, in the Philosophical Transactions, his splendid and elaborate theory of the "superadded" or "respiratory" nerves. In the ingenious essay to which we now refer, it is easy to distinguish the strong facts upon which Mr. Bell founded his theory, from the slighter instances which he trained and bent towards its support. The view which he had taken was the following:

The muscles of the face, that is to say, the nasal, labial, and palpebral muscles, receive nerves from two sources, viz. the 5th cerebral nerve, and the 7th. Muscles elsewhere in general receive nerves from one source alone, either *e.g.* from a cervical, a dorsal, a lumbar, or a sacral nerve. Now, of the two sets of nerves distributed to the muscles of the face, one, namely the 5th, had been long observed to resemble in its mode of origin the spinal nerves. It was probable, therefore, that the 5th serves in the face the same purpose as the spinal nerves in other parts. But the spinal nerves in other parts minister equally to sensation and voluntary motion. Hence it followed that the facial branches of the 5th are probably nerves of sense and voluntary motion jointly.

But what office, then, was to be attributed to the facial branches of the 7th? Let us look, the physiologist said, to the endowments of the muscles of the face, and ascertain whether they enjoy any property superadded to those which muscles elsewhere exhibit. Such a superadded endowment Mr. Bell supposed that he found, in the change of feature expressive of emotion, in the play of the nostrils in breathing, in the motion of the lips, and nose, and eyelids, in laughing and sobbing: in other words, in those movements of the features which are usually called instinctive, as opposed to such as are premeditated. And Mr. Bell concluded that the portio dura of the 7th (the superadded or respiratory nerve of the face, as he termed it,) was placed for the purpose of transmitting the instinctive impulse to muscles, already supplied with sentient and voluntary nerves from other sources, namely, from the facial branches of the 5th. Mr. Bell then proceeded to put this most original and ingenious conjecture to the test of experiment. The following are his words:

"An ass being thrown, and its nostrils confined for a few seconds, so as to make it pant and forcibly dilate the nostrils at each inspiration, *the portio dura was divided on one side of the head:* the motion of the nostril on the same side instantly ceased, while the other nostril continued to expand and contract in unison with the muscles of the chest. On the division of the nerve, the animal gave no sign of pain; there was no struggle nor effort made when it was cut across. The animal being untied, and corn and hay given to him, he ate without the slightest impediment.

"An ass being tied and thrown, *the superior maxillary branch of the fifth nerve was exposed.* Touching this nerve gave acute pain. *It was divided,* but no change took place in the motion of the nostril: the cartilages continued to expand regularly in time with the other parts which combine in the act of respiration; but the side of the lip was observed to hang low, and it was dragged to the other side. The same branch of the 5th was divided on the opposite side, and the animal let loose. He could no longer pick up his corn; *the power of elevating and projecting the lip, as in gathering food, was lost.* To open the lips the animal pressed the mouth against the ground, and at length licked the oats from the ground with his tongue. The loss of motion of the lips in eating was so obvious. that it was thought a useless cruelty to cut the other branches of the 5th."*

"From these facts," continues Mr. Bell, "we are entitled to conclude that the portio dura of the 7th is the respiratory nerve of the face; that the motions of the lips, the nostrils, and the velum palati,† are governed by its influence, when the muscles of these parts are in associated action with the other organs of respiration."‡

"We have proofs equal to experiments that, in the human face, the actions of the muscles which produce smiling and laughing, are a consequence of the influence of this respiratory nerve. A man had the trunk of the respiratory nerve of the face injured by a suppuration which took place anterior to the ear, and through which the nerve passed in its course to the face. It was observed that, in smiling and laughing, his mouth was drawn in a very remarkable manner to the opposite side."§

"*In the individual whose face was paralysed on one side during the excited state of the respiratory organs, there could be observed no debility or paralysis in the same muscles when he took a morsel into his mouth and began to chew.*"||

* Phil. Trans. 1821, p. 413.

† Mr. Bell is mistaken in supposing that the portio dura gives any branches to the velum palati.—Rev.

‡ Phil. Trans. p. 414. § Ibid. p. 416.

|| Ibid. p. 417. At the bottom of this page is a curious instance of a fact unintentionally bent to suit a theory. Mr. Bell observes, that touching the

The brilliant theory which we have thus illustrated was generally received as a most important addition to our physiological knowledge. The late Mr. SHAW went to Paris to communicate with M. MAGENDIE upon it, and to repeat the experiments upon which it was founded. M. Magendie speaks thus of the division of the 7th nerve, in reference to Mr. Bell's views:

"It remained to ascertain if the muscles to which this nerve is distributed were entirely paralysed on its division, or whether they remained still capable of performing some actions. It appeared that the act of mastication could still take place. In fine, it appeared that nothing was disturbed by the division of the portio dura but the consent of the muscles of the face with the other muscles of respiration."

M. Magendie continues,

"We have repeated these experiments at the veterinary school at Alfort, with Mr. Shaw and M. Dupuy, and the result which we have obtained agrees perfectly with that which we have related, excepting as regards the influence of the infra-orbital nerve on mastication; an influence which was not evident to us."*

In this criticism Magendie was in error. Mr. Bell was right in stating that, when both infra-orbital nerves are divided, the animal *does not* use its lips *(it seems that it cannot,)* in taking food. The experiment, however, is more satisfactory when the inferior maxillary nerves are cut as well: that is to say, when the branches of the 5th which supply the under lip are divided, as well as those which supply the upper lip.

The reader will observe that, in speaking of mastication in the preceding extracts, Mr. Bell meant to express only *the action of the lips and cheeks* in manducation, not that of the jaws, to the muscles of which no branch of the 7th is distributed, nor any nerve, indeed, that was divided or implicated in the preceding experiments.

In the midst, however, of the extensive and early celebrity which this theory obtained, it appears that some doubts were entertained by one physiologist of its correctness. Mr. MAYO was induced to consider the original reasoning upon which this theory was founded, fallacious, and the experiments by which it was supported, incomplete and inconclusive.

7th nerve in the ass convulsed the muscles of the face; but that, "by means of the branches of the 5th, *it was more difficult* to produce any degree of action in the muscles." The fact is, *it is impossible.* Irritation of the facial branches of the 5th produces no action in the muscles they supply.—REV.

* Magendie, Journ. de Physiologie, tome i. p. 387.

Mr. Mayo observed, that the muscles which have "respiratory or superadded nerves" distributed to them, are not the only muscles which seem to have two endowments. The whole frame is susceptible, although in a less degree, of instinctive excitation, as well as the countenance and throat. The muscles of the jaws, especially, which have almost as much to do in instinctive action as the muscles of the face, have no superadded nerve given to them. In other words, Mr. Mayo saw no reason why the muscles of the face, and chest, and throat, should be thought to require an additional class of nerves. He therefore proceeded to examine, patiently and rigorously, the experiments which went to establish what he surmised to be a wrong conclusion. He observed that Mr. Bell had divided the portio dura *on one side alone*, in the experiment from which he deduced that it influenced breathing, but not the prehension of food. Mr. Mayo coupled with this observation the fact that the orbicular muscle of the lips, by which the ass takes its food into the mouth, is equally supplied by the nerves of *both sides* of the face. It did not, therefore, Mr. Mayo observed, necessarily follow that, when *one* portio dura *alone* was divided, the endowment which the orbicular muscle of the lips retained was derived (as Mr. Bell thought) from the 5th nerve of the same side. It was equally likely that the result depended upon the influence of the undivided portio dura of the *other* side. But let Mr. Mayo speak for himself, and describe his own experiments.

"The portio dura was divided (in an ass) *upon both sides*. The lips immediately fell away from the teeth, and hung flaccid, and the nostril lost all movement. The muscles of the lips and nostrils seemed thoroughly paralysed.

"The infraorbital and inferior maxillary branches of the 5th (in another ass) were divided on either side, where they emerge from their respective canals. The lips did not lose their tone, or customary apposition to each other and to the teeth, but their sensibility seemed destroyed. When oats were offered, the animal pressed his lips against the vessel which contained the food, and finally raised the latter with its tongue and teeth. On pinching with the forceps the extremities nearest the lips of the divided nerves, no movement whatever of the lips ensued.

"I infer from the preceding experiments,* that, in the ass, the portio dura is a simple nerve of voluntary motion, and that the frontal, infra-orbital, and inferior maxillary nerves, are nerves of sensation only, to which office that branch of the 5th which joins

* It would have been unnecessary to quote the whole of these experiments. They were repeated and varied, but always with the same result.—Rev.

the portio dura probably contributes; *and, from preceding anatomical details, that other branches of the third division of the 5th are voluntary nerves to the pterygoid, the masseter, the temporal, and buccinator muscles.*"*

Thus was overthrown, in its strongest hold, the theory of the respiratory nerves, and a new and simple view substituted and demonstrated. Mr. Bell had taught that the infraorbital and inferior maxillary branches of the 5th are for sense and voluntary motion; the facial branches of the 7th for the transmission of an instinctive impulse.† Mr. Mayo proved that the facial branches of the 5th, above named, are exclusively nerves of sense; the facial branches of the 7th, the exclusive and common nerves of motion.‡

One point, we see, has escaped our notice. How happens it that, when the labial branches of the 5th are alone divided, the animal does not use its lips in taking food? Mr. Mayo, in the essay which we have just quoted, most happily and satisfactorily resolved this difficulty. He pointed out that the fact adverted to is a necessary consequence of the loss of sensation in the lips; referring his readers to the history of cases of anæsthesia, in which a parallel phenomenon is uniformly recorded. When a person has lost sensation in his hands (their muscular power remaining,) they are useless to him, unless he directs another sense to guide and regulate their efforts: as long as a patient thus afflicted looks at what he sustains in his hand, so long the object is secure; the instant that he turns his head away, the object drops from his grasp. No muscular effort can be sustained without a sense to guide it.

* Mayo's Anatomical and Physiological Commentaries, Part I. p. 112.—London, 1822.

† Suppose a writer, in describing the circulation, were to adopt the phrase of "the vessels which convey the blood from the heart to all parts of the body;" should we not be justified, to avoid such unnecessary circumlocution, in inferring and asserting that he meant the *arteries*? Mr. Bell mentions all those actions which must be deemed *instinctive*, but prefers avoiding the use of this term, which we, as we think, more philosophically, employ.—Rev.

‡ In looking carefully over Mr. Bell's first essay upon the Nerves, (Philos. Trans. 1821, p. 418,) we find the following passage, which, *taken alone*, would seem to show that he thought one division of the 5th nerve merely a sentient nerve; the 7th, in that region, an exclusively motor nerve. Coupled, however, with the context, it is evident that the facts which we have to quote, if difficult to be reconciled to his theory, did not tend to shake his faith in it. "I divided," says Mr. Bell, "the branch of the 5th pair which goes to the forehead, in a man, at his urgent request, on account of the tic douloureux: there appeared no paralysis of the muscles of the eyebrow. But, in an individual where an ulcer and abscess, seated anterior to the tube of the ear, affected the superior branch of the respiratory nerve, the eyebrow fell low, and did not follow the other, when the features were animated by discourse or emotion."

Such are the conclusions at which Mr. Mayo arrived, in the researches which he set on foot avowedly in order to sift the theory proposed by Mr. Bell; and these conclusions of Mr. Mayo's, Mr. Bell has subsequently published as his own, having, as it appears to us, substantially abandoned his theory of the respiratory nerves,* and adopted a truer view, without (we regret to say,) acknowledging to whom he is indebted for the correction.

The next step in the history of these inquiries is the discovery by Magendie of the functions of the double roots of the spinal nerves. In the autumn of 1822, Magendie published, in his Physiological Journal, the details and results of his decisive experiments. He divided the *posterior* roots of the spinal nerves in a young animal; upon which, sensation was found to be lost, while the power of motion remained. He divided the *anterior* roots in another: motion was lost, but feeling remained. Thus Magendie proved the anterior roots or fasciculi of the spinal nerves to be voluntary or motor nerves, and the posterior fasciculi to be nerves of sensation. We attribute this great discovery entirely to Magendie.† It is childish in any other physiologist to advance a claim to it; and, although the pupils of Mr. Bell and Mr. Mayo may take pleasure in tracing how near each of these physiologists had approached to the discovery, we, with the caution of practised critics, look only to the date of the respective publications, in which each new fact is announced; and allot to each author, according to *that date*, his claim to originality.

Pursuing this method, the next experiment upon which we fall is the following by Mr. Shaw. Mr. Shaw divided the 5th nerve immediately after its exit from the cranium. He found "that, upon this, the jaw fell, and that the mus-

* See Mr. Bell's Summary of the Uses of the Nerves. Phil. Trans. 1823, p. 300.

† "While referring to matters of alleged plagiarism, M. Magendie being accused, as well as Mr. Mayo, of this offence, it may perhaps assist the cause of the former, if I were to state the fact that, on the 2d September, 1822, M. Magendie informed me, while sitting beside him at a séance of the Institute in Paris, that he had recently found by experiments (very difficult to adopt) that, the anterior roots of the spinal nerves being divided, the parts supplied by these nerves lost their powers of motion, and retained their sensibility; and that, when the posterior roots were divided, the parts supplied by these nerves lost their sensibility, but retained their motion. As to the question of M. Magendie's originality, and Mr. Bell's priority in the discovery of the separate functions of the spinal columns and nervous roots, I will not venture to give any positive opinion. M. Magendie's communication *appeared to me*, at the period quoted, to be new as far as it went, although I was aware that Mr. Bell had *paved the way* to the important facts becoming developed of the separate functions allotted to different portions of the nervous system."—*Extract from Mr. Broughton's Letter, Med. Gazette, June 6, 1829.*

cles of that side were powerless."* To this experiment we are not inclined to attach much value. Mr. Mayo had *previously* made the observation, that the branches of the 5th distributed to the muscles of the jaws *must* be motor nerves, inasmuch as the muscles of the jaws receive nerves from no other source. But our late much respected friend, Mr. Shaw, in the essay in which his experiment is published in this Journal, appears to us to have seen *beyond the facts* which his experiment proved. He certainly compares the 5th to the spinal nerves with more precision than Mr. Bell had done. But, on the other hand, it must be admitted that he no where states, in so many words, *what part* of the muscular branches of the 5th he supposes to be fasciculi of motion, or voluntary nerves.†

Upon *this* inquiry Mr. Mayo, it appears, was bent at the time when Mr. Shaw made the experiment which we have described. "Towards the close of last summer," observes Mr. Mayo, in the second part of his Anatomical Commentaries, published in July 1823, "I endeavoured to trace the final distribution of the ganglionless portion of the 5th nerve in the ass, and I succeeded in making out that it furnishes those branches, which are exclusively distributed to muscles. This dissection I have repeated four times, and in an adjoined drawing have represented the fact as existing in the ass. I have since ascertained that, in the human body, precisely the same distribution exists."‡

Let us give equal credit to Mr. Mayo and Mr. Shaw upon this question. Both appear to have pursued a similar anticipation, but by different methods. Mr. Shaw made his experiment; Mr. Mayo, his dissection. If Mr. Shaw's experiment was not thoroughly to the point, Mr. Mayo, in *his* subsequent researches, had to correct his own anatomy, as will appear in our next citation.

The present stage of the inquiry brings us to consider the paper by Mr. Bell, of which the title stands at the head of this article. Its object appears to be twofold: first, to point out the general distribution of the ganglionless portion of the 5th; secondly, to establish, by reference to the supposed distribution of one of its branches, a partial illustration of the reasonableness of the "respiratory" theory. But it

* London Medical and Physical Journal, October 1822, p. 349.

† How vague Mr. Shaw's impressions must still have been respecting the ganglionless portion of the 5th is evident from the plate of the origins of the nerves which he subsequently gave in this Journal for December 1822.—Rev.

‡ Anatomical and Physiological Commentaries, Part II. p. 9. July 1823.

has now for several years been matter of general knowledge that the ganglionless portion of the 5th is distributed to the muscles of the lower jaw, as their nerve of motion. *This* point, therefore, required no further elucidation. With reference to the *second* point, it almost raises a smile, even upon the grave countenance of a reviewer, to have to deal with a statement seriously propounded in the Philosophical Transactions for 1829, the substance of which Mr. Mayo published in 1822, and, having found it to be erroneous, published the correction of his mistake in 1823. But let us give the words of Mr. Bell.

"I prosecuted," observes Mr. Bell,* "with more interest the ramus buccinalis labialis. And nobody, I presume, will doubt that the distribution of this division confirms the notions drawn from the anatomy of the trunk, not only that the 5th nerve is the manducatory nerve as belongs to the muscles of the jaws, but also that it is distributed to the muscles of the cheek and lips, to bring them into correspondence with the motion of the jaws. Let us take in illustration the articulation of the bones. In the joints the muscles are attached to the capsular membrane in such a manner as to draw it from between the bones, and adapt it to the degree of flexion of the joint. If the cheek were a passive membrane, like the capsule of a joint, it would have required some such mechanical connexion with the jaw, or its muscles, as might have drawn it from between the teeth in the motions of mastication. But, being a muscular part, to bring it into just relation with the motions of the teeth, it must have an accordance through nerves, and act in sympathy: relax when the jaws are apart, and contract when they are closed. I think, therefore, we may perceive why a branch of the motor nerve of the muscles of the jaws sends a division to the muscles of the cheek and to the angle of the mouth."

From this statement it appears that Mr. Bell supposes the ramus buccinalis labialis to be a motor nerve to the buccinator. Such, too, it appears Mr. Mayo thought it in 1822. But, in 1823, Mr. Mayo gives a different account of the matter.

"I mentioned," says Mr. Mayo, "that I concluded that other branches of the 5th nerve, from their distribution to the pterygoïd, masseter, temporal, and buccinator muscles, are voluntary nerves. This conclusion involved a trifling error: the pterygoïd, masseter, and temporal muscles, are indeed exclusively supplied by the 5th, and therefore, without doubt, the branches so distributed are voluntary nerves; but the buccinator receives branches from the portio dura as well, and I have found subsequently that pinching

* Phil. Trans. 1829, p. 325.

the branch of the 5th, which perforates that muscle, produces no action in it."*

Again, in page 10 of the same essay, Mr. Mayo says, "In the preceding Number I have mentioned that the division of the portio dura of the 7th nerve paralyses the muscles of the face. Now, the buccinator muscle is intermediate between the cutaneous muscles of the lips and nostrils and the powerful muscles moving the jaw; and it is somewhat difficult to determine, after the division of the portio dura, whether the muscle in question be paralysed or not; but the question may be decided by pinching in succession, in the dead ass, that branch of the 5th which perforates the buccinator, and then the trunk of the portio dura. While the former experiment is unattended with any effect, the latter produces a distinct spasm of the buccinator, as well as of the other muscles about the lips and nostrils."

Mr. Mayo's account of the nervus buccinalis labialis is completed in his system of Dissections, published in 1825. In this volume there is an excellent figure of the distribution of the third division of the 5th; and the following remarks upon the branch of it which we are now considering, occur in the explanation of the plate:

"The nervus buccinatorius is represented as giving off two branches to the pterygoïdeus externus, and one to the temporal muscle, and finally turning over the margin of the former towards the buccinator muscle. I have satisfied myself, by repeated dissections, that *the portion of the nerve which passes onwards to the buccinator muscle and membrane of the mouth, does not contain any filaments from the smaller (the ganglionless) fasciculus; those elements of the buccinator nerve which are derived from the latter source are consumed in its temporal and pterygoïd branches.* Since ascertaining the preceding anatomical facts, (facts already quoted about the ganglionless part of the 5th,) I find that they were known to Palletta, with the exception of that marked in italics."†

When we consider the evidence thus laid before our readers, and couple it with the facts that Mr. Mayo was the first to divide (in another experiment) the fifth nerve, *within the cranial cavity*, by which he established that the common sensibility of the eye depends on the 5th, and that the motion of the iris is independent of that nerve;‡ and that Mr. Mayo has shown the portio dura of the 7th and the 5th to rise together, and to be but *one nerve*,§ (the portio dura and

* Mayo's Anat. Comment. Part II. p. 8. When a *motor* nerve is pinched with forceps, in an animal recently killed, the muscles which it supplies are convulsed. This does not happen when a sentient nerve distributed to a muscle is irritated.—Rev.

† Mayo's Course of Dissections, p. 167, 1825.

‡ Mayo's Anat. Comment. Part II. p. 5.

§ Mayo's Outlines of Physiology, and Plates of the Brain.

the ganglionless part of the 5th having together the same relation to the ganglionic portion of the 5th which the anterior roots of the spinal nerves have to the posterior roots,) we feel that to him belongs the merit of having done most towards elucidating the functions of these nerves, and that his name will be associated in physiological science with the history of the 5th and 7th nerves, as that of Magendie with the history of the spinal nerves.*

Let us now turn to Mr. Bell's intermediate papers in the Philosophical Transactions, and consider the rest of his system of the respiratory nerves. We are compelled to be brief. There is not here, indeed, much temptation to enter into long discussion. Mr. Bell, misled by a theory, appears to us generally wrong upon all the points in which he advances what is new. We shall shortly advert to the leading ones.

In the first place, the pneumo-gastric nerve, the glosso-pharyngeal, and the seventh, do not arise, as Mr. Bell supposed, from a peculiar column of the medulla oblongata, but pass through the surface and great part of the substance of the medulla oblongata, to rise, as Mr. Mayo has shown, not from a tract apart, but *conjointly and together with the 5th and 6th and auditory nerves, from the floor of the 4th ventricle.*† In their origin, (however dissimilar the functions of these nerves are shown to be experimentally,) they have a strange agreement. In the second place, the spinal accessory nerve is met with in birds, as Mr. Mayo exhibited in his recent lectures before the College of Surgeons, and therefore has probably some other object in human beings, than to associate the action of the sterno-cleido-mastoïdeus and trapezius with the muscles of the chest in breathing. The spinal accessory rises, besides, from the back part of the medulla spinalis, not from the fore part, as Mr. Bell has represented it.‡ And in the third place, and finally, it is perfectly ridiculous to describe the phrenic nerve and the

* The reviewer has great pleasure in finding his judgment of the relative claims of Messrs. Bell and Mayo confirmed by the opinion of Mr. Broughton. This talented surgeon, who is also distinguished as a physiologist by his researches upon digestion, and more recently upon respiration, repeated, after Mr. Mayo, the experiments which we have narrated. The reader will find, in letters in the Medical Gazette, May 9th and June 6th, 1829, that Mr. B. attributes to Mr. Mayo the discovery of the uses of the facial branches of the 5th and 7th, and likewise the use of the ganglionless part of the 5th.

† Magendie has fallen into a remarkable error in tracing an imaginary origin for the 6th at the root of the anterior pyramid.—See *Anatomie des Systemes Nerveux*, par Magendie et Desmoulins; Plate 13. Paris, 1825.

‡ Bell's Exposition of the Nerves, p. 55.

external thoracic nerve as having any distinguishing peculiarity, in their common and spinal origin, from the other branches of the axillary and cervical nerves.

With equal brevity must we despatch Mr. Bell's remarks upon the eye.*

In the first place, the observation that the eyeball is raised during sleep, is Soemmering's, not Mr. Bell's.

In the second place, the inferior oblique muscle of the eye directs the axis of the eye upwards and outwards, not upwards and inwards, as Mr. Bell supposes.

In the third place, our seeing objects erect by means of inverted images depends upon a law of vision, most ingeniously illustrated by Mr. Mayo's experiment of compressing the retina, which is totally independent of, and unconnected with, any sense of exertion and muscular activity, to the suggestions of which Mr. Bell refers this phenomenon. Nothing, again, can be wilder than to contrast the straight and oblique muscles of the eye, the first as voluntary, the second as involuntary (instinctive?) muscles: the distribution of the nerves precludes any such arrangement; and an examination of the comparative anatomy of the optic muscles clearly proves the correctness of the common notions on this subject. In fish, reptiles, birds, and mammalia, as Mr. Mayo displayed in his College lectures, the obliqui are uniformly placed as antagonists to the recti; the recti always draw the eye backward, the obliqui always sling the eye forward. The recti individually turn the eye upwards, downwards, outwards, or inwards, as well as retract it: the obliqui rotate the eye, as well as draw it forward.

The best account of the functions of the nerves is contained in Mr. Mayo's "Outlines of Human Physiology." The subject is here stripped of theoretical disguise, and is treated with that lucid brevity which marks Mr. Mayo's style. We have before strongly recommended Mr. Mayo's treatise to our readers; once more we point out its value. The simplicity of truth never delights so much as when the mind has been previously bewildered and fatigued with brilliant, but vague and delusive, speculations.

* Philos. Trans. 1823, p. 175.

*A Treatise on Neuralgic Diseases, dependent upon Irritation of the Spinal Marrow and Ganglia of the Sympathetic Nerve.* By THOMAS PRIDGEN TEALE, Member of the Royal College of Surgeons in London, of the Royal Medical Society of Edinburgh; senior Surgeon to the Leeds Public Dispensary, &c. —8vo. pp. 120. Highley, London, 1829.

IT is to the industry of modern pathologists that we are indebted for the information we possess respecting many of those diseases which are dependent upon organic changes or functional derangements of the spinal marrow. Hippocrates, Celsus, Aretæus, &c. were aware that convulsions and palsy of the limbs might arise from lesions of this important organ. This prominent fact could not escape their notice, and it was almost the only one with which they were acquainted in reference to the subject. By his clinical observations, Frank threw much light upon this interesting inquiry.* He advanced much further than any of his predecessors in his investigations into this most important part of pathology; and, if he failed to prove, he at least showed it was highly probable, that many painful and obscure diseases, attacking remote parts of the body, arose from injury or irritation of the spinal marrow. We do not mean to assert that Frank entered upon this investigation without assistance from previous writers. By various authors, and Hoffman in particular, it had been suggested that affections of the spinal marrow might have a greater share in the production of many diseases than had previously been supposed. Frank, however, did more than throw out suggestions: he appealed to his practical observations to prove the solidity of his doctrines; but still he, in general, wanted that convincing evidence which dissection alone could have afforded.

To approach nearer our own day, we may mention the work of Dr. Ollivier, which contains a valuable body of information.† Upon this subject the researches and experience of Dr. Abercrombie have also been given to the profession;‡ and to this able and most useful writer we should be grateful for many ingenious observations and illustrative cases, which tend to show how various and perplexing are the symptoms which frequently owe their origin to disease or derangement of the spinal cord. The interesting

* De Vertebralis Columnæ in Morbis Dignitate. Delect. Opuscl. Med. vol. ii. 1792.

† Traité de Moelle Epiniere, et de ses Maladies, tome ii. Paris, 1827.

‡ Abercrombie on Diseases of the Brain and Spinal Cord. 2d Edition 1829.

essay of Dr. and Mr. Griffin, of which the first part is contained in our present Number, must not be passed over without honourable mention. It is a valuable contribution to our knowledge of the various functional disturbances which often arise from irritation of the spinal cord.

But we must proceed to the analysis of the work before us.

The term Neuralgia, Mr. Teale observes, which was originally employed to designate certain affections of nerves attended with severe pain, has of late, with great propriety, been extended from its original and literal signification, to many other morbid affections of nerves which are not characterised by pain, but by some other perverted state of their functions.

We cannot admit that any term can be thus wrested from its true signification "with great propriety." To avoid such an abuse of language, it would be better, upon the principle *de malis minimum*, to add a more accurate expression even to our already over-stocked vocabulary, which would correctly include the diseases it was intended to define. If a classical patient, free from pain, were told that his malady was "neuralgic," he would attribute the vicious diction either to the philological or the professional ignorance of his physician. In the above more extended signification, however, the term is not unfrequently employed, and the number of "neuralgic affections" is thus greatly increased.

Mr. Teale believes that the difficulty and embarrassment which have attended the diagnosis and treatment of these affections have principally arisen from mistaken views of their pathology.

"They have too often been regarded as actual diseases of those nervous filaments which are the immediate seat of the neuralgia, instead of being considered as symptomatic of disease in the larger nervous masses from which those filaments are derived: hence the treatment has too frequently been ineffectually applied to the seat of neuralgia, instead of being directed to the more remote and less obvious seat of disease." (*Introduction,* p. 2.)

It is now generally admitted as a pathological axiom, that disease of the larger nervous masses, as the brain and spinal marrow, is not so much evinced by phenomena in the immediate seat of disease, as in those more remote parts to which the nerves arising from the diseased portion are distributed. In the more severe forms of disease, this principle is readily admitted and recognised.

"When, for instance, one half of the body shall have lost its sensibility, and the corresponding muscles their power of action, the skin and the muscles are not regarded as the seat of disease, but the brain is immediately referred to. In the slighter forms of disease of the brain and spinal marrow, such as do not completely obliterate, but merely impair or pervert the functions of the nerves, such as do not paralyse the sentient and muscular powers of the part, but produce weakness, tremors, spasms, &c. in the muscular system, and numbness, pricklings, pains, and other morbid feelings in the nerves of sensation, this important principle, which as strictly obtains as in the former instance, is too often entirely overlooked; and a numerous class of complaints, of very frequent occurrence, are regarded as nervous or spasmodic diseases of the part affected, instead of being considered as actual diseases of that portion of the brain or spinal marrow from which the nerves of the part are derived." (*Ib.* p. 3.)

The same pathological principle Mr. Teale conceives is equally applicable to the sympathetic system of nerves; and, although it may be difficult to establish this opinion by actual experiment, yet he thinks it may be rested upon a well-grounded analogy, which will justify us in regarding the nervous masses of the ganglionic system as bearing the same relation to the nerves derived from them, as the large nervous masses of the cerebro-spinal system bear to their respective nerves.

"Influenced by such considerations, I have, for a few years, been in the habit of treating many of these nervous affections as diseases of some portion of the spinal marrow or ganglia; and have been still further confirmed in my opinion by the frequent and almost uniform co-existence of tenderness on pressing some portion of the vertebral column, and the circumstance of the tender portion of the spine being in the particular situation where the nerves of the affected part originate." (*Ib.* p. 4.)

In corroboration of the opinions he advances, Mr. Teale quotes freely from Dr. Brown,* Dr. Darwall,† and Mr. Player;‡ each of whom has recently published excellent essays upon the subject.

The symptoms of irritation of the spinal marrow consist in an infinite variety of morbid function of the nerves of sensation and volition, which have their origin in the spinal marrow. The parts in which these morbid functions are exhibited, of course, bear reference to the distribution of the spinal nerves.

* On Irritation of the Spinal Nerves, by Thomas Brown, M.D. (Glasgow Med. Journal, May 1828.)

† Observations upon some forms of Spinal and Cerebral Irritation, by John Darwall, M.D. (Midland Med. and Surg. Reporter, May 1829.)

‡ Quarterly Journal of Science, vol. xii. p. 428.

"The morbid states of sensation include every variety, from the slightest deviation from the healthy sensibility of any part to the most painful neuralgic affections on the one hand, and to complete numbness or loss of feeling on the other; including pains which may be fixed or fugitive, or darting in the direction of the nerves, prickling and tingling sensations, a sense of creeping in the skin, of cold water trickling over it, and numerous other states of perverted sensation, of which words are inadequate to convey a description. In the muscular system we find weakness or loss of power, tremors, spasms or cramps, and sometimes a tendency to rigidity.

"These symptoms sometimes exist in so slight a degree, that the patient considers them unworthy of notice, and only admits their existence when particular inquiry is made respecting them: the only complaint which he makes, being of an unaccountable sense of weakness and inability of exertion. In other cases the tremors have excited alarm; sometimes the neuralgic pain in the scalp, or the fixed pain in the muscles, particularly when it occurs in the intercostal muscles, has suggested the idea of serious disease in the brain or in the lungs; and when the pain is seated in the muscles of the abdomen, a fear that some organic disease of the abdominal viscera has taken place harasses the mind of the patient. The muscular weakness, in some cases tending to paralysis, often suggests the fear of apoplexy, or paralysis from cerebral disease.

"The affection is often of very protracted duration, undergoing alternate variations, from the sanative powers of the constitution and the different exciting causes of disease. There are many individuals in whom the complaint has existed, in varying degrees of intensity, for a series of years, without its real nature having been suspected; the patients and their medical attendants having regarded it throughout as a rheumatic or a nervous affection." (P. 13.)

In this complaint, tenderness in the portion of the vertebral column which corresponds to the origin of the affected nerves, is generally evinced by pressure. The symptoms, of course, vary according to the particular part of the spine which is affected, and bear reference to the distribution of the spinal nerves.

"When the upper cervical portion of the spinal marrow is diseased, we frequently find neuralgic affections of the scalp; the pain strikes in various directions over the posterior and lateral parts of the head; sometimes the twigs in the neighbourhood of the ear, sometimes those which ascend over the occiput to the superior part of the scalp, are more particularly the seat of the complaint; the nervous twigs distributed to the integuments of the neck are occasionally affected, the pain darting across the neck to the edge of the lower jaw, and sometimes encroaching a little upon the face. These neuralgic diseases frequently assume an intermittent form, the paroxysms generally occurring in the evening. A stiff neck, or impaired action of the muscles moving the head, frequently attends the affection of the upper cervical portion of the spinal marrow; and occasionally the voice is completely lost, or suffers alteration, and the act of speaking is attended with pain or difficulty.

"Irritation of the lower cervical portion of the spinal marrow gives rise to a morbid state of the nerves of the upper extremities, shoulders, and integuments at the upper part of the thorax. Pains are felt in various parts of the arm, shoulder, and breast; sometimes the pain takes the course of the anterior thoracic branches of the brachial plexus, occasionally the pain is fixed at some point near the clavicle, scapula, or shoulder-joint, at the insertion of the deltoid, or near the elbow, or shoots along the course of some of the cutaneous nerves. Frequently one or both of the mammæ become exquisitely sensible and painful on pressure, and some degree of swelling occasionally takes place in the breast, attended with a knotty and irregular feel, when the neuralgic pains have existed a considerable time in that part. Prickling and numbness, tingling and creeping sensations, are often felt in the upper extremities; and also a sensation of cold water trickling over the surface. On rubbing the hand over the part affected, a soreness is frequently felt, which is described as not merely situated in the integuments, but also in the more deep-seated parts. In the muscular system are observed most frequently a weakness of the upper extremities, sometimes referred particularly to the wrists; tremors and unsteadiness of the hands; also cramps and spasms, of various degrees of intensity. Occasionally there is an inability to perform complete extension of the elbows, the arm appearing restrained by the tendon of the biceps; pain and tightness being produced in this part when extension is attempted beyond a certain point. As far as I have observed, the pains and other morbid feelings in the upper extremities and chest are felt more frequently and more severely on the left than on the right side." (P. 15.)

When the upper dorsal portion is affected, in addition to various morbid sensations similar to those in the extremities, there is often a fixed pain in some part of the intercostal muscles, and perhaps tenderness on pressure. The lower dorsal half of the spinal marrow being the seat of the irritation, the symptoms are again modified. Frequently there is a sensation of a cord tied round the waist; soreness along the cartilages of the lower ribs; pains, fixed or fugitive, in the parietes of the abdomen.

From a similar affection of the lumbar and sacral portion of the spinal cord, there often arises soreness in the scrotum, spasms, tremors, and weakness of the lower extremities. A recumbent position is frequently of service.

"This irritation, or subacute inflammatory state, of the spinal marrow, is not necessarily connected with any deformity of the spine, or disease in the vertebræ. It may coexist with these, as well as with any other diseases; but it so repeatedly occurs without them, that they cannot be regarded as dependent upon each other. Where, however, inflammation and ulceration of the vertebræ or intervertebral cartilages exist, it is probable they may predispose to, and in some instances act as, an exciting cause of, an inflammatory state of the nervous structures which they contain; for we not unfrequently find inflammatory affections of the vertebræ in conjunction with symptoms of irritation of the spinal marrow. But these two affections, although coexisting, bear no regular relation to each other; and, during the progress of the vertebral disease, the affection of the nervous structures is subject to great changes and fluctuations. The local remedies employed for arresting the disease in the bones often alleviate the affection of the spinal marrow, at the very commencement of the treatment, long before the vertebral disease is suspended; but, as the neighbouring inflammation in the bones appears to predispose or excite the nervous mass which they contain to disease, relapses of the nervous affections are repeatedly occurring during the whole course of the complaint." (P. 18.)

The lateral curvature and excurvation of the spine are not considered by Mr. Teale to have any necessary connexion with spinal irritation. If the pressure and stretching of the nerves produced by the curvature were the cause of the nervous symptoms, they would continue as long as the deformity remained, which is not the case.

*Treatment.* When the different neuralgic symptoms which have been enumerated can be traced to this morbid state of some portion of the spinal marrow, the treatment that ought to be pursued is readily decided upon.

"Local depletion by leeches or cupping, and counter-irritation by blisters to the affected portion of the spine, are the principal remedies. A great number of cases will frequently yield to the single application of any of these means. Some cases, which have even existed several months, I have seen perfectly relieved by the single application of a blister to the spine, although the local pains have been ineffectually treated by a variety of remedies, for a great length of time. A repetition of the local depletion and blistering is, however, often necessary after the lapse of a few days, and sometimes is required at intervals for a considerable length of time. In a very few obstinate cases, issues or setons have been thought necessary; and where the disease has been very unyielding, a mild mercurial course has appeared beneficial." (P. 20.)

The diet and general health of the patient are, of course, to be attended to. A recumbent position is frequently beneficial; but experience has taught Mr. Teale that it is not essential, unless there should be actual disease of the vertebræ. Stimulating liniments are recommended, if there be any fear of a relapse.

To illustrate these observations, a series of cases is given of neuralgic affections of various parts of the body, arising from spinal irritation, or slight inflammation. We select the following as examples.

"Mrs. B., æt. fifty-three, mother of a large family, represents herself as having been severely afflicted with rheumatism during the greater part of her life. She now suffers from pain in the neck and head, pains about the clavicles, difficulty in moving the arms, which feel fixed at the shoulder-joints. The pain in the neck and between the shoulders is fixed and constant, being nearly the same both day and night: it is a little alleviated by supporting the back against a chair. There are also darting pains extending from the cervical portion of the spine upwards over the occiput, and downwards across the neck and over each shoulder. Both arms are affected with aching pains over their whole extent, and with a sense of soreness on pressing or rubbing the skin; prickling sensation, cramps, and numbness in the forearms, hands, and fingers. Difficulty in moving the arms, and in using her fingers in sewing or knitting. Frequent sudden 'twitching' pains in the neck, arms, and trunk; occasional pains in the abdominal muscles, relieved by recumbency. No affection of the lower extremities; appetite poor; no fever; no cough or difficulty of breathing; catamenia ceased about six years ago.

"She has always considered the disease to be rheumatism, and has tried a great variety of remedies usually employed in that disease, but without much benefit.

"Tenderness in the two lower cervical and six upper dorsal vertebræ.

"Leeches were directed to be applied to the tender portion of the spine, and on the following evening a blister to the same part. Recumbency was also recommended.

"The blister produced an unusual degree of inflammation in the skin, which continued several days, and was accompanied with considerable fever. During the febrile state the neuralgic symptoms were rather aggravated, but, as the fever subsided, they gradually disappeared.

"On the 29th of December I took leave of her, as she was then perfectly well: she felt a degree of muscular power, particularly in the upper extremities, which she had not been accustomed to for several years. She was quite free from pain.

"June 20th, 1829. Since the last report she has enjoyed good health, with the exception of occasional returns of the pain during winter, which were so slight as to produce but little inconvenience, and were soon relieved by leeches and the application of turpentine liniment to the spine. These last attacks were attended with flatulence." (P. 25.)

The next case, of "neuralgia of the mamma, or irritable breast," deserves especial attention.

"Mrs. ——, æt. forty-eight, but without having experienced any change in the catamenia, of a healthy appearance, and mother of a large family, had suffered about seven years from a painful affection of the left breast. On examination, it was found to be exquisitely sensitive to the slightest touch; it was somewhat increased in size, and irregularly indurated, having a knotty feel, and an obscure sense of tumors, as if the glandular structure were enlarged at different parts. The integuments and cellular substance between the breast and clavicle, and towards the axilla, were thickened. There was a constant sense of uneasiness in the part, but her chief sufferings arose from its highly sensible state, which constantly exposed her to pain from the irritation of her dress, or any accidental contact. Her spirits were depressed, and an apprehension that the disease would prove cancerous, although she was repeatedly assured of the contrary, was a source of great anxiety. Leeches, evaporating lotions, and warm fomentations, had been employed, and medical treatment had been particularly directed to the digestive organs: these means were occasionally productive of slight alleviation, but never of permanent benefit. The complaint varied in degree, being sometimes less severe for a few weeks, without any obvious cause for the temporary amendment.

"Whilst in this state, (September 1827,) she became subject to pains in the scalp, and vertigo, attended with flatulence. These symptoms directed my attention to the spine, which, on examination, was found to be tender in several parts. The most painful vertebræ were the second cervical, the seventh cervical, and two upper dorsal. Leeches were applied to these parts, with considerable relief to the pains in the scalp and vertigo. Since that time she has been occasionally in the habit of applying leeches, a blister, or a sinapism, of her own accord, when there has been any return of uneasiness in the head.

"On making inquiry (August 10th, 1829,) respecting the complaint in the breast, of which I had not heard any mention for several months, she tells me that from the time of her commencing the treatment by local applications to the spine, the affection of the breast has disappeared. The pain and swelling are removed, and the breast resembles the other in every respect.

"The circumstance of finding a portion of the spine tender, and the removal of the tenderness by suitable remedies being unexpectedly accompanied with relief of the fulness and pain in the breast, could not fail to produce a powerful impression on my mind, and to excite a suspicion that this irritable affection of the breast was a neuralgia of that part dependent upon disease of the spinal marrow." (P. 26.)*

Mr. Teale next devotes a few pages to the consideration of certain neuralgic affections of the heart and stomach, which he is disposed to attribute to irritation of the ganglia of the sympathetic nerve. To show that this conjecture is probably correct, he refers to various physiological experiments, from which, he presumes, it may be inferred,

"That painful affections of the nerves of the heart, lungs, and stomach, are not seated in the filaments of the pneumo-gastric nerve, since this nerve is not a nerve of sensation, and therefore cannot be the seat of pain; consequently, that they must be seated in the filaments of the sympathetic.

"That the action of the blood-vessels and muscular viscera is dependent upon the sympathetic, and consequently that irregularities in the action of these involuntary muscles may, with much greater probability, be referred to disease in the sympathetic than in the cerebro-spinal system.

"That, as digestion has been observed to take place in some instances after the division of the eighth pair, and that it proceeds in animals which have not this nerve distributed to the stomach, it is evident that some other system of nerves (the sympathetic) exerts a considerable influence in digestion, and consequently that disease in the sympathetic may disorder or interrupt the digestive process." (P. 63.)

Mr. Teale is apparently not aware of the fact stated by Mr. Mayo, "that the fibrils which it (the pneumo-gastric nerve) distributes to the larynx, and pharynx, and œsophagus, are nerves of *sensation* and motion."† From the cautious manner in which Mr. Teale very judiciously offers his suggestion of the *probability* that irritation of the sympathetic ganglia gives rise to the various neuralgic symptoms he describes, it is evident that he has formed a correct estimate of the great uncertainty that yet hangs over the functions of this intricate part of the nervous system. He confesses that, "in the absence of direct evidence from dissection, the precise nature of these affections of the spinal marrow and ganglia must, to a certain degree, remain conjectural."

As a proof that the ganglia are occasionally the seat of disease of such intensity as to produce permanent alteration in their structure, and that the symptoms produced were

* Sir Astley Cooper gives a chapter upon the "irritable tumor," in which many instructive remarks are made, both as to the symptoms and diagnosis of this disease. (Illustrations of theDiseases of the Breast, ch. ix. p.76.)—Rev.

† Outlines of Physiology, 2d Edition, p. 338.—Rev.

*principally exhibited in the remote organs* to which the nerves of the affected ganglia were distributed, some cases recorded by Lobstein are referred to.* Mr. T. relates ten cases of neuralgic affections of the heart, stomach, and other parts of the body, in none of which did the patient complain of any pain in the spinal column, although, upon a careful examination, tenderness of the vertebræ was discovered. In some of these instances the true cause of the symptoms had been overlooked by other practitioners, and the treatment adopted had consequently failed. The experience of Mr. Teale enabled him to detect the real source of mischief, and, by the application of leeches and blisters to the affected part of the spine, occasionally a recumbent position, and proper attention to the general health, the patients were relieved.

In conclusion, Mr. Teale offers some remarks on angina pectoris. He gives a brief account of the different opinions that have been held respecting the pathology of this malady.

"Numerous cases have occurred, presenting the characteristic signs of angina pectoris, in which a perfect recovery has taken place, which could scarcely have been possible if any considerable organic change had existed in the structure of the heart. Many other cases of angina, which have proved fatal, and have been inspected after death, have not exhibited any traces of diseased structure in that organ; and repeated instances of ossification of the coronary arteries have been met with, in which the symptoms of angina were not present. From these circumstances we must conclude that the organic changes in the structure of the heart are not essential to the disease, and that although they frequently co-exist with angina, yet they are not the cause of those symptoms to which that name has been assigned. We must then look to some other source for the explanation of the phenomena." (P. 99 )†

Many writers of repute have attributed the most distressing symptoms of angina pectoris to disturbance of some portion of the nervous system;‡ but this appears to have been regarded more as an accidental or collateral circumstance, the affection of the heart being regarded as the principal disease. Mr. Teale is fully convinced that it is

* London Medical and Physical Journal, March 1824.

† Mr. H. Watson relates a case of very extensive ossification of the coronary arteries, in which there was no symptom of thoracic disease. (Medical Commun. vol. i. p. 234.) Dr. Latham (College Trans. vol. iv. p. 278,) describes two cases of enlarged liver, in which all the genuine symptoms of angina pectoris were observed. Both patients died suddenly.—Rev.

‡ Dr. Wall, Med. Trans. vol. iii. Dr. Fothergill, Med. Obs. and Inq. vol. v. 1776. Dr. Johnstone, Mem. Med. Soc. vol. i. Treatise on Angina Pectoris, by W. Butter, M.D. 1791. M. Desportes, sur l'Angine de Poitrine, Paris, 1813. Laennec, Traité de l'Auscultation Mediate, &c. 2d Edit.

to the nervous system we must look for the seat of this disease;

"but the great error which has been committed by those who have assigned to angina pectoris a seat in the nerves, consists in their having overlooked the pathological fact to which I formerly alluded, namely, that when any of the nervous masses, as the brain, spinal marrow, or ganglia, are the seat of disease, the morbid phenomena are not so much exhibited in the masses themselves, as in the parts to which the nerves arising from them are distributed. * * The treatment has also been conducted with reference to such pathological views: blisters, issues, and other remedies have been applied by the earlier writers to the neighbourhood of the heart or stomach; most frequently, however, without much benefit." (P. 103.)

Mr. Teale has been induced to refer the various groups of symptoms which have been described as angina pectoris to an affection of some portion or portions of the spinal marrow, and of the corresponding ganglia of the sympathetic, by the following considerations:

"1. The fact, as I have before observed, that most of the morbid phenomena exhibited in the extreme filaments of nerves, are seldom owing to disease in the nerves themselves, but to an affection of the nervous mass from which they are derived.

"2. The co-existence of pain on pressing some portion of the spine with the symptoms constituting angina pectoris; and the correspondence of the painful part of the spine with the particular symptoms which are present; namely, tenderness in the lower dorsal portion of the spine in conjunction with the stomach affection, constriction, &c., and tenderness in the cervical spine, with pains in the arms, breast, and shoulders, and palpitations.

"3. The relief obtained by local antiphlogistic measures to the spine; for instance, to the lower dorsal portion when the stomach is affected and there is constriction, and to the cervical portion when there is an affection of the arms and palpitations." (P. 107.)

Three cases are related, which appear to support the opinions of the author. In each, local tenderness of the vertebræ was detected, and the symptoms were relieved by the application of blisters, leeches, and stimulating liniments, to the part.

Mr. Teale is anxious that he may not be thought desirous of advocating a theory of uniform infallibility, or a practice invariably successful. "Disappointments will occasionally occur, and failure must sometimes be encountered."

We have been much interested in the perusal of this little work, and we believe there are few practitioners who may not derive instruction from the pathological doctrines and practical hints it contains.

*An Experimental Inquiry into the Laws which regulate the Phenomena of Organic and Animal Life.* By George Calvert Holland, m.d. Bachelor of Letters of the University of Paris; formerly Senior President of the Hunterian Medical Society, and President of the Royal Physical Society of Edinburgh.—8vo. pp. 462. Edinburgh, Maclachlan and Stewart; and Simpkin and Marshall, London, 1829.

It would be impossible within the limits of an ordinary review to follow the author of the volume before us, step by step, through the close critical examination to which he subjects various physiologists, who have before entered the same field of investigation. Indeed, if we had the space, we should hardly have the courage to step in, as intermediate critics, between different experimental physiologists, who from the very same experiments arrive at the most discrepant inferences. A great part of the work is occupied in attempting to shew that John Hunter, Wilson Philip, and Dr. Edwards, of Paris, and other physiologists, have established erroneous conclusions, from the various experiments which they have instituted, for the purpose of clearing up different physiological perplexities. Neither can we do full justice to the many original speculations Dr. Holland enters into, in reference to the laws which regulate the phenomena of organic and animal life. Some of his most important corollaries we shall notice, and especially those which bear upon practical points; without, however, entering into the detailed arguments upon which his doctrines are established, for they can only be duly estimated by an attentive perusal of the volume itself. We shall confine ourselves, therefore, to a succinct account of the leading objects of the work, which will be sufficient to convey to our readers a general impression of its character. In a very neat introduction, Dr. Holland points out the danger of being too indulgent, either to the experimentalist or theorist. The former seldom commences his practical inquiries without having some preconceived view or principle to establish or refute, and, whatever be the nature of the conclusions, he is apt to seize and apply with avidity such only as are consonant with his own opinion.

Many pages are occupied in considering the source of animal heat; and, since the completion of the work, the author tells us an idea has struck him which appears sufficient, in conjunction with principles to which he refers, to explain it satisfactorily.

"The explanation I shall propose goes far to support the doctrine of Black, in which the increase of heat is attributed to chemical changes in the lungs. It is now, I believe, almost universally allowed, that the arterial is warmer than the venous blood, and it is more than probable that this result depends on chemical action. By taking into consideration, that a small quantity only of the air within the lungs is at any one moment deteriorated, and, still further, that the left ventricle contracts 70 or 80 times per minute, in order to propel the arterial blood which is transmitted by the lungs, we shall have reasons sufficiently ample to account for the possibility of these organs bearing such changes, and for the ease with which the system is supported in an equable temperature. If the body be supposed to possess 30 pounds of blood, and the heart to transmit at each contraction two ounces, and to contract 75 times per minute, we shall find that the whole mass of blood will pass through the lungs once every three minutes, or twenty times per hour. As it has been proved by direct experiment that the blood acquires at least one degree of heat in passing through the lungs, it necessarily follows, at this moderate calculation, that the system will receive 20 degrees of heat in an hour, or 240 degrees every twelve hours. If the respiration be accelerated, and the contractions of the heart be increased to 100, the mass of the blood will circulate through the thoracic organs in one fourth less time than is stated above, and consequently the temperature will be augmented one fourth: the increase of one degree, instead of being repeated every three minutes, will be repeated every two and a quarter minutes.

"According to the doctrine of Crawford, the evolution of heat is confined to the capillaries distributed throughout the body; but the present explanation of the manner in which the system acquires 20 degrees per hour, or 240 every twelve hours, is unfavorable to such an opinion, as it proves that the lungs transmit an immense quantity of sensible heat to the body, a quantity, it is highly probable, sufficient for every organic necessity.

"From this view of the chemical changes in the lungs, it is apparent that the various internal parts of the system will possess, as nearly as possible, the same degree of animal heat. The blood which the left ventricle sends out at one contraction, is calculated to supply the deficiencies incurred by an equal portion which is returned to the right auricle; and as the whole circle of circulation is completed in two or three minutes, there can scarcely be a perceptible difference in the temperature of the different parts of the system." (*Introduction*, p. 20.)

The three first chapters are chiefly devoted to an examination of the physiological inferences, drawn from experiments, by Wilson Philip, upon the subjects of animal heat and digestion. Dr. Holland endeavours to prove that these inferences are incorrect; but, while we admit that he has displayed a certain degree of argumentative ingenuity, we cannot assign to him the merit of having overthrown the doctrines he attacks. In the fourth chapter are some interesting observations on the distribution of the blood, at different ages and seasons. Dr. Holland draws the following conclusions.

"1. That the blood in all young animals is generally diffused through the system, on account of the internal necessities making little demand upon this fluid; and that the character of this distribution is changed in proportion to the developement of these necessities.

"2. That, at the maturity of the animal frame, the internal organs are more vigorous than at any other period of life; and that, as the natural or diseased action of these is augmented, if unaccompanied by fever or exercise, the blood in all cases is determined to them in greater quantity than natural, either maintaining the regular internal circulation, or extending this to a state of aberration.

"3. That, at the decline of the powers of life, the blood is more internal in its circulation than at any other period, from the concurrent influence of the previous gradual changes tending to promote this effect, and from the imperfection of those functions essential to renew its qualities and facilitate its motion." (P. 115.)

The succeeding chapter is on the "temperature at different ages;" and in the sixth, the manner in which the system is adapted to the influence of cold is considered. Upon one subject, the best authorities are opposed to Dr. Holland: he imagines that insane persons are peculiarly insusceptible to extreme cold, and in proof of this opinion, he remarks that,

"There are, indeed, several cases on record, in which individuals are stated to have escaped from confinement when affected with insanity, during the greatest severity of winter, without the protection of even their ordinary dress, and who were afterwards taken, and found to have been exposed for many hours to the bitter wind of the season, without suffering from the exposure."(P.158.)

A few instances are insufficient to establish a general principle. Dr. Burrows, the best practical writer of the day, on insanity, declares that there is no mistake which has inflicted such incalculable evils upon the inmates of lunatic asylums, as the erroneous belief that they are particularly insusceptible to extreme cold. So far from this being true, he states that they are generally very obnoxious to either extreme of temperature.* Dr. P. S. Knight, also, observes that "lunatics, like all other invalids, cannot bear cold so well as persons in health: in fact they are always chilly and seek warmth, and their animal powers

* Commentaries on Insanity, by G. M. Burrows, M.D., p. 288.

are below the healthy standard, frequently indeed greatly reduced, and their inability to resist the effects of cold is in proportion."* Dr. Holland, indeed, appears to have founded his opinion upon those cases of insanity in which the patient keeps himself warm by constant exercise. But this fact does not shew that lunatics in particular are at all insensible to cold. The sane, as well as the insane, when in active bodily exercise, are equally insusceptible to the depressing influence of diminished temperature; and, if the mind be powerfully excited, many other external sensations are unheeded.

In the next chapter the author offers a few brief remarks on the rapidity of hibernating animals. This subject is more fully discussed in Dr. Fleming's work on Zoology.† The continuance of life during the period of hibernation, in animals, is thus succinctly yet satisfactorily accounted for:

"No part of Nature subject to the laws of organization is indebted for its existence to the excited activity of its functions, but to the observance of those intimate relations subsisting between the powers which add and those which subtract. Whenever the former predominate over the latter, life then displays its exuberance, the plant throws out its leaves or expands its flowers, and the energy of the animal frame is concentrated to strengthen old or develope new functions, or to excite disease; and when the latter become superior to the former, the plant droops, and the animal decays. But if these powers are equally diminished, as vegetation is in winter, or as happens to animals in a state of torpidity, it is almost impossible to prescribe boundaries to their duration." (P. 165.)

The means by which the system is enabled to bear a temperature much superior to that of the body; the influence of disease on the production of heat, and the function of the eighth pair of nerves; the influence of narcotics on the generation of animal heat, and on the digestive powers; and the causes which influence the action of the heat, are the subjects which are next considered. The thirteenth chapter embraces a more practical discussion, that of palpitation; the general and organic causes which produce this affection are inquired into.

"Palpitation of the heart occurs very frequently in the constitution of the female at two very different periods of life. On the developement of the uterine functions, the circulation of the blood undergoes great changes; new demands are made, and the regular

* Observations on Derangement of the Mind, by PAUL SLADE KNIGHT, M.D. 1827, p. 123.

† Vol. II. p. 45.

course of the vital fluid is directed to other organs. But these important alterations are not immediately established, and, until they are, disorders in the circulation, disturbing the motion of the heart, are extremely liable to occur. When these organs are fully formed, the delicacy of their functions is subject to the influence of many circumstances, all of which act powerfully on the distribution of the blood. At the latter period of life, the demands of these parts of the system are diminished. The blood, which has for a series of years been determined here to supply the activity of functions unessential to life, is now no longer required. It is therefore the intention of nature to diffuse the quantity appropriated to these functions equally throughout the system; but the attainment of this object is occasionally opposed, or rendered imperfect by a variety of causes calculated to derange the sanguiferous system.

"The application of cold has the tendency to determine the blood upon the internal organs; and if palpitation be the consequence of its influence, this arises from an overcharged state of the lungs." (P. 285.)

The fifteenth and sixteenth chapters are on the "Physiology of passion," and the "nature of the vital principle." These are subjects upon which we could not touch without entering into a wide and very unsatisfactory field of speculation.

Chapter 17. On sympathy. In this chapter the author "attempts to expose many important errors, either committed or supported by physiologists of eminence;" and at the same time proposes opinions, that appear to him better calculated to solve the most essential phenomena of sympathy, and assist our indications of practice. Sympathy, however produced by the different states of the body, Dr. Holland divides into four classes: sanguineous, sensorial, nervous, and nervo-sensorial.

"1. By the term sanguineous sympathy, I allude to affections produced and propagated by changes in the nature, quantity, and circulation of the blood.

"2. By sensorial sympathy, I mean certain effects originating in sensations or states of the mind, subsequently propagated through the medium of nerves: an example of this division is the production of syncope and vomiting from a mental cause.

"3. Nervous sympathy is the propagation of certain effects accomplished by nerves alone, as in risus sardonicus, arising from irritation or inflammation of the diaphragmatic nerve communicated to the seventh pair of the face.

4. Nervo-sensorial is a term employed to explain phenomena, which result from the irritation of a nerve or nerves, of which the brain takes cognizance, and which afterwards transmits its impressions to other organs of the body. Vomiting is frequently an instance of this reaction." (P. 400.)

The last chapter treats of the general action of emetics on the system, and contains some remarks on their efficacy in chronic and acute diseases.

From the sketch which we have given of this work, it will be seen that it is almost entirely occupied in canvassing the doctrines of previous physiological writers. In support of his peculiar opinions, Dr. Holland frequently argues with much talent and ingenuity.

---

### COLLECTANEA.

Floriferis ut apes in saltibus omnia libant,
Omnia nos, itidem, depascimur aurea dicta.

